# Recognizing Solo Jazz Dance Moves Using a Single Leg-Attached Inertial Wearable Device

**DOI:** 10.3390/s22072446

**Published:** 2022-03-22

**Authors:** Sara Stančin, Sašo Tomažič

**Affiliations:** Faculty of Electrical Engineering, University of Ljubljana, 1000 Ljubljana, Slovenia; saso.tomazic@fe.uni-lj.si

**Keywords:** motion recognition, motion analysis, inertial sensors, 3D accelerometer, 3D gyroscope, solo jazz, dancing

## Abstract

We present here a method for recognising dance moves in sequences using 3D accelerometer and gyroscope signals, acquired by a single wearable device, attached to the dancer’s leg. The recognition entails dance tempo estimation, temporal scaling, a wearable device orientation-invariant coordinate system transformation, and, finally, sliding correlation-based template matching. The recognition is independent of the orientation of the wearable device and the tempo of dancing, which promotes the usability of the method in a wide range of everyday application scenarios. For experimental validation, we considered the versatile repertoire of solo jazz dance moves. We created a database of 15 authentic solo jazz template moves using the performances of a professional dancer dancing at 120 bpm. We analysed 36 new dance sequences, performed by the professional and five recreational dancers, following six dance tempos, ranging from 120 bpm to 220 bpm with 20 bpm increment steps. The recognition *F1* scores, obtained cumulatively for all moves for different tempos, ranged from 0.87 to 0.98. The results indicate that the presented method can be used to recognise repeated dance moves and to assess the dancer’s consistency in performance. In addition, the results confirm the potential of using the presented method to recognise imitated dance moves, supporting the learning process.

## 1. Introduction

Dancing is an engaging physical activity that requires high levels of body control, skill, and physical fitness. In addition, dancing is an activity cultivating the capacity for creative expression. Following the rhythmic structure, the dancer, especially the professional, expresses his or her interpretation of music by selecting, performing, and assembling various moves in dance sequences.

The main motivation of the research presented is to provide a mechanism for dance move recognition in performed sequences. In general, it has already been reported that dancers can greatly benefit from various assistive technologies [1,2,3,4,5]. A dance move recognition technology would benefit dancers engaged in the learning process. Namely, as one learns to dance, he or she tends to imitate performances demonstrated by instructors. These imitations are visually assessed, usually using a mirror. In addition, instructors provide their students with feedback on how their performances compare to the ideal template. Providing such feedback is usually more challenging in group sessions, in which more students engage in the learning process at the same time. A technological solution would benefit both the student and the instructor and would support performance assessment and progress monitoring.

More experienced dancers would also benefit. Recognising dance moves would enable further investigation and comparison of the sequences of moves, supporting overall creativity evaluation, individual style classification, and observations of differences when dancing alone, in pairs and/or in the presence of an audience. Additionally combining the recognised dance moves with information about the accompanying music would illuminate how the dancer interprets and responds to music. Relying on a dance move recognition technology during the creative process of producing a choreography, a professional dancer would also be able to create a transcript of the assembled moves for later reference.

A segment of dance motion analysis approaches made so far relies on signals obtained from optical motion capture systems, incorporating multiple cameras watching the stage and usually requiring reflective markers to be positioned on the subject’s body [6,7,8,9]. In [6], the authors present a framework for capturing the posture of the dancer’s body. Using the body posture parameters, signals are temporally segmented, motion patterns are extracted, and motion animation in generated. In [7], the authors present dance pose sequences similarity estimation.

Another segment of approaches relies on signals obtained from a Kinect device [1,2,5,10,11,12]. The Kinect device incorporates a depth camera together with a standard video camera into a single device, distinguishes human body parts and joints, and estimates their position and orientation. In [1], the authors present a novel framework for the real-time capture, assessment, and visualisation of ballet dance movements. The dance sequence captured is segmented and cross-referenced against a library of gestural components performed by a teacher. Recognition performance was evaluated on a database of six isolated basic ballet positions recordings made by a teacher and a student. Reported average recognition rates are between 90.5% and 99.5%. Bharatanatyam [2] and a salsa dancing coach application [5] have also been developed. In [10], another Kinect-based system is presented enabling Korean pop (K-pop) motion classification by extracting statistical features, reducing dimensionality with Principal Component Analysis (PCA) and Linear Discriminant Analysis (LDA) and incorporating extreme learning machine. Skeletal joint angles for 200 movement types obtained for four dancers were considered and the reported maximum classification rate is 96.5%. In [11], the authors analysed data on Greek traditional dance postures with the goal of extracting robust summaries and providing end users or dance experts a concise and meaningful abstract of dance movements.

Although valuable results have been reported, using equipment located in the surrounding area, either one or more video cameras or a Kinect device, limits the practical aspect of the methodology, making it difficult or altogether impossible to dance in the ballroom. Moreover, processing video signals is computationally intensive, and although the underlying technology and algorithms are steadily improving, computer vision is still limited by lighting and clutter constraints and subtraction of dynamic background.

The presented method capitalises on the benefits of small and lightweight microelectromechanical (MEMS) inertial sensors. Over the past few years, it has been consistently demonstrated that these sensors are an efficient tool in the broader research area of human motion analysis [13,14,15,16,17,18,19,20,21,22,23,24,25,26,27,28,29]. Their characteristic light weight, small size, low power consumption, portability, ease of use, and low cost pave the way for their omnipresence and indispensability in motion analysis.

Most of the research conducted in this field concerns daily activity recognition [13,14,15,16,17,18,19], gait analysis [20,21,22], gesture recognition [23], and sports activity performance assessment for activities such as golf, tennis, baseball, swimming, and skiing [24,25,26,27,28,29]. One of the first wearable systems for capturing dance gestures were developed by the MIT Media Lab [30,31], and various assistive solutions have been presented since [3,4,32,33,34,35,36,37,38,39]. In this context, wearable devices are usually used to detect dancer’s steps and provide them with feedback [3,4,32] or enable the dancer to interact or generate sounds [33,34,35]. In [36], a three-axis inertial measurement unit (IMU) is positioned on the subject’s right forearm and used to analyse one specific hip hop arm move. In particular, the aim is to divide the motion into phases, predict which phase is executed, and give feedback to the subject. In addition, accelerometers are used to estimate torso tilt [37] and assess performance [38] of basic routines in classical ballet.

We focus our analysis on the solo jazz dance style, a rhythmical and playful solo dance in which the dancer depicts jazz music through one’s one movement. Solo jazz evolved through the first half of the 20th century to include elements of both African and European dance and features the vocabulary and steps of the vernacular jazz tradition. To enable the dancer to follow the jazz song rhythmical structure, as a rule, a single solo jazz move is performed following eight music beats. The music beat is considered as the smallest time interval between two successive notes. Dance moves comprise various motion elements, performed in a specified order. Besides steps, motion elements can be, for example, kicks, taps, and jumps. The variety of motion elements and the order of their execution brings a pleiad of predefined, authentic solo jazz dance moves. Due to this variety, solo jazz fits perfectly into our dance move recognition research polygon.

In general, in dancing, the speed of move execution represents the dance tempo and is directly related to the musical tempo of the song. It is measured as the number of steps, or other motion elements, a dancer performs in a minute. As a rule, solo jazz is usually danced to jazz music with tempo anywhere between 120 and 250 beats per minute (bpm). Tempos between 140 and 160 bpm are considered medium. Tempos above 220 bpm are considered fast and generally prove to be too high for the recreational dancers to execute with accuracy and ease.

Aiming to provide ease of use, with the smallest amount of sensing equipment, our dance move recognition methodology relies on a single wearable unit, comprising a MEMS 3D accelerometer and gyroscope, positioned on the dancer’s leg, and enables dance move recognition, regardless of the device orientation and dance tempo. The methodology presented considers that different moves have different signal shapes in the time domain and that these shapes can be used as dance move fingerprints. Furthermore, since position and attitude estimates obtained using inertial sensors are known to be characterised by different inaccuracies, especially drift, the methodology resides on the shapes of the original acceleration and angular velocity signals instead.

The methodology presented is a continuation of our previous work and features solo jazz dance tempo estimation, as presented in [39]. In addition, the specific technique presented in [20], used for defining a wearable device orientation-invariant coordinate system using the 3D accelerometer signals acquired during walking, is adapted and applied in the research presented in this article.

The remainder of this article is organised as follows. In Section 2, we present the materials and methods used for creating the dance move template database, recognition, and experimental validation. In Section 3, we present and discuss the results. Finally, in Section 4, we summarise our findings and draw conclusions, implying further research directions. In all the subsequent sections, we use the following notation rules: large bold letters denote matrices, small bold letters denote vectors, and large or small italics denote scalars.

## 2. Materials and Methods

### 2.1. Data Acquisition

#### 2.1.1. Materials

We captured dance motion using a single mbientlab MetaMotionR (MMR) wearable device [40], including a MEMS 3D accelerometer and gyroscope with respective measurement ranges ± 16 g and ± 2000°/s, placed directly above the dancer’s right leg ankle. We hypothesised that analysing the motion of a single leg is sufficient to distinguish between various dance moves. An alternative solution would be to place the device on the dancer’s lower back, since leg motion of high-quality dance performances is usually initiated from the pelvic region, torso, or even the shoulders. However, preliminary investigations have shown that this significantly reduces the recognition performance of the designed solution. The micro-position and orientation of the device are arbitrary.

We set the sampling frequency to 200 Hz, which proved to be sufficient for the problem at hand using empirical evidence. The 3D accelerometer and gyroscope together provided 6 inertial signals, each representing one variable in a 6D space.

In addition, a software metronome with an established beat frequency error of 1 bpm was used to simulate a steady quarter note music tempo and dictate the tempo of dancing.

#### 2.1.2. Measurements

Six female dancers participated in the study—five recreational dancers (age: 33 ± 5 years, height: 163 ± 5 cm) with over three years of experience in solo jazz dancing and one professional (age: 35 years, height: 164 cm).

Altogether, *I* = 15 authentic solo jazz moves were considered: (1) Tackie Annie, (2) Fall of the log, (3) Kicks, (4) Half break, (5) Struttin’, (6) Savoy kick, (7) 20s Charleston, (8) Knee slaps, (9) Fishtails, (10) Apple Jacks, (11) Boogie back, (12) Boogie forward, (13) Crazy leg, (14) Cross step, and (15) Shorty George. For an informative overview of how these moves are performed, the reader is referred to various sources available online, e.g., [41]. Each move was performed on an 8-beat basis.

Measurements were performed in two sessions. Following the obtained instructions, participants attached the wearable device above their right leg ankle, voluntarily setting its micro-position and orientation.

The first measurement session was conducted only for the professional dancer. The dancer performed several consecutive repetitions for each of the 15 considered moves, as consistently as possible, following a fixed reference tempo *υ_ref_ =* 120 bpm, dictated by the metronome. For each move, the dancer started dancing from a still position. We refer to the obtained set of six inertial signals as the learning sequence.

The second measurement session was conducted for all six participants. Each was given the task of performing 5 repetitions of each of the 15 considered moves in a prescribed order. The recreational dancers were instructed to mimic the professional’s execution of moves as closely as possible. This task was repeated for 6 different dance tempos, ranging from 120 bpm to 220 bpm with 20 bpm increments. For each dancer, we obtained 6 sets of inertial signals. We refer to these 36 sets as test sequences.

All measurements were supplemented with video recordings. The study was conducted in Ljubljana, Slovenia. It followed the Code of Ethics of the University of Ljubljana, which provides guidelines for studies involving human beings and is in accordance with the Declaration of Helsinki. All participants gave written informed consent.

### 2.2. Signal Processing Overview

**Figure 1:**
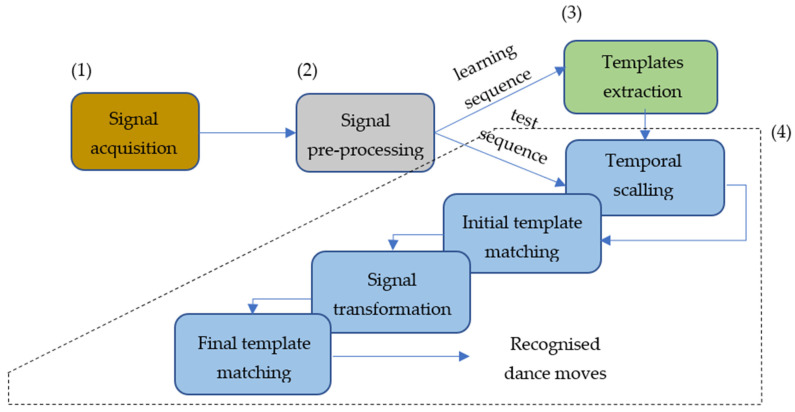
Dance move recognition processing workflow.

The implemented signal processing workflow is presented in Figure 1. For each acquired sequence (1), signal pre-processing (2) is applied. The pre-processed learning sequence is used to extract template moves and create the template database (3), following a semi-automatic correlation-based segmentation procedure. The pre-processed test sequences are analysed and searched for template moves (4), according to the following steps:

4.1. Dance tempo estimation and signal temporal scaling, achieved using a bank of enhanced comb filters as presented in [39];
4.2. Initial template matching, performed on a sliding correlation basis, using the magnitudes of the temporally scaled acceleration and angular velocity;
4.3. Signal transformation to the templates’ coordinate system; and
4.4. Final template matching, performed again on a sliding correlation basis, but by using the acceleration and angular velocity 3D projections of on the template coordinate system axes instead of their magnitudes.

For both the initial and final template matching, correlation is used as the only feature for recognition. In the following, all the components of the proposed method are presented in detail. All signal processing was executed offline in the MATLAB 2021 environment [42].

### 2.3. Signal Pre-Processing

Following the calibration procedure presented in [43], we compensated all signals of the learning and test sequences for sensor inaccuracies. Since the device itself does not provide outputs at exactly equidistant time samples, we interpolated and decimated the acquired signals as necessary, considering the associated measurement timestamp values, to provide for uniform sampling at exactly 200 Hz. To remove motion artefacts and noise, we applied a low-pass filter with a cut-off frequency *f_co_* = 50 Hz and finally performed downsampling to *f_s_* = 100 Hz, obtaining 3D acceleration and angular velocities at equidistant time samples T = 1/*f_s_* = 0.01 s.

### 2.4. Templates’ Database

#### 2.4.1. Template Extraction

Using all *N_s_* acquired samples of the pre-processed learning sequence, we formed two *N_s_* × 3 signal matrices, $A_{s}$ and $\Omega_{s},$ representing 3D acceleration and angular velocity, respectively. Columns of these two matrices are equal to the respective signal projections on the device-intrinsic coordinate system axes *x*, *y*, and *z*, while rows represent the time samples. The specific orientation of axes *x*, *y*, and *z* in a reference coordinate system, defined by the set position of the wearable device, is arbitrary. We further combined $A_{s}$ and $\Omega_{s}$ in a common *N_s_* × 6 signal matrix **S**:

(1) $$S=\left[\begin{array}{cc} A_{s} & \Omega_{s} \end{array}\right].$$

Columns of **S** are equal to the 3D acceleration and angular velocity *x*, *y*, and *z* projections, while rows represent the time dimension. Comprising the inertial signals of the entire learning sequence, matrix **S** comprises all performed repetitions of all considered dance moves, used for creating the templates’ database.

We extracted the comprised dance move repetitions by partitioning **S** into submatrices in the vertical dimension. We achieved this by estimating the repetitions’ onsets and offsets, for each 1 ≤ *i* ≤ *I* = 15 dance move separately, following a semi-automatic, correlation-based segmentation procedure as follows. First, since for each dance move, the consecutive repetitions are performed from a still position, we defined the onset of the first repetition by visual inspection of the six inertial signals and the accompanying video. We denote the matrix **S** row index corresponding to this onset as *n_0_*_._

For defining the offset of the first repetition, we consider, given the known reference dance tempo *υ_ref_*, the expected length, expressed as the number of samples *N*_T_, of a single dance move. Since each considered solo jazz move is performed on an 8-beat basis, at *f_s_* = 100 Hz sampling and precise 120 bpm dancing (*T_beat_* = 0.5 s), the expected length is *N*_T_ = 8 × 0.5 s × 100 Hz = 400 samples. Since dancing is rarely this precise, we allow for a 2% length deviation and set the expected length to be in between 0.98 × *N*_T_ = 392 and 1.02 × *N*_T_ = 408 samples. Each length from this interval, i.e., *N_n_* = *N_0_* + *n*, where *N_0_* = 392 and 0 ≤ *n* ≤ 0.04 × *N*_T_ = 16, gives a candidate for the first repetition offset, *n_0_* + *N_n_* − 1.

Since the repetitions are consecutive, for each offset candidate for the first repetition, *n_0_* + *N_n_*_−_ 1, there is a single onset candidate for the second repetition, *n_0_* + *N_n_*. Finally considering both repetitions to be equal in length gives a single offset candidate for the second repetition, *n_0_* + 2*N_n_* − 1. For each n, we obtained one candidate pair of the first two repetitions of a move, represented with adjacent, same-size *N_n_* × 6 submatrices of **S**.

We extracted and standardised to zero mean and unit standard deviation these two submatrices and performed column-wise vectorisation, obtaining, for each *n*, two 6(*N_0_* + *n*) long column vectors. Pursuing the highest similarity, for each candidate pair *n*, we calculated the correlation coefficient between these two vectors. This calculation, supported with implementation equations, is described in detail in Appendix A.2.

We set the first and the second repetition of the considered move to be equal to the repetitions of the candidate pair with the highest correlation coefficient. We finally extracted all possible additional repetitions of the particular move on an equal correlation-based search basis using the onset of the last determined repetition as the new *n_0_*.

Due to slight variations in execution duration, we unified in length all extracted repetitions by temporal scaling to the exact expected length *N*_T_ = 400 samples. For each move *i*, we composed a set of consistent repetitions, eliminating repetition outliers, determined by visual inspection. We averaged this set column-wise, obtaining finally, for each template move *i*, an associated single *N*_T_ × 6 matrix $T_{i}.$ Denoting the first three columns, representing the 3D acceleration, with A*_i_* and the second three, representing 3D angular velocity, with $\Omega_{i}^{},$ for each 1 ≤ *i* ≤ *I* = 15, we can write:

(2) $$T_{i}^{}=\left[\begin{array}{cc} A_{i} & \Omega_{i}^{} \end{array}\right].$$

The 15 matrices $T_{i}$ of the acceleration and angular velocity projections on the device intrinsic coordinate system axes *x*, *y*, and *z* represent our database of template moves. The specific orientation of the axes *x*, *y*, and *z* in the reference coordinate system, defined by the arbitrarily set position of the wearable device during the learning sequence acquisition, defines the coordinate system of the templates.

In addition to **T***_i_*, for each move, *i* we also calculated an *N*_T_ × 2 matrix $\bar{\bar{T_{i}}},$ having the first and second column equal to the magnitudes of the 3D acceleration and angular velocity, respectively. Each *k*-th row of $\bar{\bar{T_{i}}}$ is obtained according to:

(3) $$\begin{array}{cc} {\bar{\bar{T_{i}}}}_{k,*}^{} & =\left[\begin{array}{cc} \left\|A_{i\;k,*}\right\| & \left\|\Omega_{i\;k,*}^{}\right\| \end{array}\right]\\ & =\left[\begin{array}{cc} \sqrt{A_{i\;k,1}^{2}+A_{i\;k,2}^{2}+A_{i\;k,3}^{2}} & \sqrt{\Omega_{i\;k,1}^{2}+\Omega_{i\;k,2}^{2}+\Omega_{i\;k,3}^{2}} \end{array}\right]. \end{array}$$

In (3), * denotes all columns.

#### 2.4.2. Templates Similarity Measures

To estimate the similarity between template moves, we used two correlation-based measures as follows. Firstly, we compared the template moves along the acceleration and angular velocity magnitudes. To achieve this, we standardised the columns of $\bar{\bar{T_{i}}}$ to zero mean and unit standard deviation for each 1 ≤ *i* ≤ *I* = 15 move and performed column-wise vectorisation of the resulting matrix. For each move, we obtained a 2*N*_T_-long vector of magnitudes. For each pair of template moves *i* and *j*, 1 ≤ *i,j* ≤ *I* = 15, we calculated the correlation between the associated vectors as a function of cyclical shift 0 ≤ τ < *N*_T_ of the acceleration and angular velocity vector parts. We define the maximum value of this correlation for each *i,j* pair of template moves, denoted with $r_{\bar{\bar{T_{i}}}\bar{\bar{T_{j}}}},$ as the magnitudes’ similarity. This calculation is supported in detail with implementation equations in Appendix A.3.

We also compared the template moves along the acceleration and angular velocity 3D projections. We standardised the columns of **T_i_** to zero mean and unit standard deviation for each 1 ≤ *i* ≤ *I* = 15 move and performed column-wise vectorisation of the resulting matrix. For each move, we obtained a 6*N*_T_ -long vector. For each pair of template moves *i* and *j*, 1 ≤ *i,j* ≤ *I* = 15, we again calculated the correlation between the associated vectors as a function of cyclical shift 0 ≤ τ < *N*_T_ of the projections vector parts. We define the maximum value of this correlation for each *i,j* pair of template moves, denoted with $r_{T_{i}T_{j}},$ as the projections’ similarity. This calculation is supported in detail with implementation equations in Appendix A.4.

In the same way as the correlation coefficient calculated along one dimension, $r_{\bar{\bar{T_{i}}}\bar{\bar{T_{j}}}}$ and $r_{T_{i}T_{j}}$ can take any value from the range [−1,1], where 1 indicates identical moves, 0 orthogonal moves, and –1 opposite moves. High $r_{\bar{\bar{T_{i}}}\bar{\bar{T_{j}}}}$ values suggest common leg activation patterns, regardless of the actual direction of motion in 3D. On the other hand, high $r_{T_{i}T_{j}}$ suggests that two moves also match with respect to the direction of the executing motion. Considering this, the similarity measure $r_{T_{i}T_{j}}$ is expected to have a higher discriminative potential. Note that by performing standardisation column-wise, equal weight is given to each dimension of comparison, *x*, *y*, and *z*, regardless of the intensity of motion along that dimension.

### 2.5. Dance Move Recognition

#### 2.5.1. Dance Tempo Estimation and Temporal Scaling

For each pre-processed test sequence, we first estimate the dance tempo *υ_est_*, using a method based on multiple resonators, implemented with enhanced comb feedback filters, as presented in [6].

We temporally scale all acquired inertial signals of the sequence by a factor of *υ_ref_*/*υ_est_* to obtain a test sequence with dance moves that match the template moves in tempo. We denote with *N_X_* the final length of the test sequence.

Using all *N_X_* samples, we form two *N_X_* × 3 signal matrices of 3D acceleration and angular velocity, denoted with $A_{X}$ and $\Omega_{X},$ respectively. The columns of these two matrices correspond to the respective inertial signal projections on the intrinsic coordinate system axes of the wearable device. Since the orientation of the wearable device is different for each measurement session, these axes and the axes of the coordinate system of the template moves are generally not aligned.

We further form a common *N_X_* × 6 test sequence signal matrix **X**:

(4) $$X=\left[\begin{array}{cc} A_{X} & \Omega_{X} \end{array}\right].$$

Columns of **X** correspond to the acceleration and angular velocity 3D projections while rows represent the time dimension.

We also form an *N_X_* × 2 matrix of acceleration and angular velocity magnitudes $\bar{\bar{X}}.$ Each *k*-th row of $\bar{\bar{X}}$ is calculated according to:

(5) $$\begin{array}{cc} \bar{\bar{X}}{\;}_{k,*}^{} & =\left[\begin{array}{cc} \left\|A_{X\;k,*}\right\| & \left\|\Omega_{X}{}_{\;k,*}^{}\right\| \end{array}\right]\\ & =\left[\begin{array}{cc} \sqrt{A_{X\;k,1}^{2}+A_{X\;k,2}^{2}+A_{X\;k,3}^{2}} & \sqrt{\Omega_{X\;k,1}^{2}+\Omega_{X\;k,2}^{2}+\Omega_{X\;k,3}^{2}} \end{array}\right]. \end{array}$$

In (5), * denotes all columns.

#### 2.5.2. Initial Template Matching

We search the time-scaled test sequence for template moves by applying sliding correlation-based template matching, initially considering the acceleration and angular velocity magnitudes. For the template *i* and the test sequence, these are comprised in matrices $\bar{\bar{T_{i}}}$ (3) and $\bar{\bar{X}}$ (5), respectively.

For each template move 1 ≤ *i* ≤ *I* = 15, we first standardise the columns of $\bar{\bar{T_{i}}}$ to zero mean and unit standard deviation. By vectorising the standardised matrices column-wise, for each template move *i*, we obtain a 2*N*_T_-long vector $\bar{\bar{t_{i}}}$ of the acceleration and angular velocity magnitudes.

We further apply a 2D sliding window of size *N*_T_ × 2 to the test sequence signal matrix $\bar{\bar{X}}$. For each 1 ≤ *n* ≤ *N_X_* − *N*_T_ + 1, we extract an *N*_T_ × 2 submatrix of $\bar{\bar{X}}$, i.e., $\bar{\bar{X}}{\;}_{n:n+N_{T}-1,*}$. After standardising and vectorising $\bar{\bar{X}}{\;}_{n:n+N_{T}-1,*}$ we obtain a 2*N*_T_-long vector of the associated acceleration and angular velocity magnitudes. We then calculate the correlation coefficient between this vector and $\bar{\bar{t_{i}}}$. Sliding the 2D window vertically, we repeat this process for all *n* and obtain *N_X_* − *N*_T_ + 1 correlation coefficients. We store the results for each template move *i* in a vector denoted with $r_{\bar{\bar{T_{i}}}\bar{\bar{X}}}.$ This calculation is supported in detail with implementation equations in Appendix A.5.

Indicating high similarity between $\bar{\bar{T_{i}}}\;\mathrm{and}\;\bar{\bar{X}}\;$, the local maximums of $r_{\bar{\bar{T_{i}}}\bar{\bar{X}}}$ reveal the possible onsets of template move *i* executions in the test sequence. For each dance move *i*, we identify the local maximums of $r_{\bar{\bar{T_{i}}}\bar{\bar{X}}}$ that exceed a certain threshold value, denoted with *r_min_*. We define the indices of these local maximums as move *i* onsets in the sequence. We further define the associated values of $r_{\bar{\bar{T_{i}}}\bar{\bar{X}}}$ local maximums as the recognition confidence.

After searching through the entire sequence for all template moves, we performed a final correction in the sense of false positives detection and elimination: if any two onsets of moves were closer than 0.7 *N*_T_, we discarded the one with the lower recognition confidence.

#### 2.5.3. Signal Transformation

To transform the signals of the test sequence from the device intrinsic to the coordinate system of the templates, we first identify from all onsets found in the previous step, for all *i* and for all *n*, the one with the highest recognition confidence. This onset defines the strongest matching pair: for *i* and *n*, for which the recognition confidence is the highest, the magnitude matrices of the template and subsequence, i.e., $\bar{\bar{T_{i}}}$ and $\bar{\bar{X}}{\;}_{n:n+N_{T}-1,*},$ respectively, match the most. We denote the corresponding matrices of the acceleration 3D projections with **A_TM_** and **A_XM_** and the combined matrices of the accelerations and angular velocity 3D projections with **T_M_** and **X_M_**.

Since this template–subsequence pair represents the same move, but in different coordinate systems, it is reasonable to assume that a rotation that aligns them can be defined. Moreover, if we determine such a rotation, we can apply it to the entire test sequence, obtaining the acceleration and angular velocity 3D projections in the coordinate system of the templates.

To define this rotation, we adapted and applied an orientation-independent transformation, as presented in [20]. The original procedure estimates a wearable device orientation-independent coordinate system by calculating three orthogonal unit vectors from the 3D accelerometer signals, acquired using a smartphone in the front pocket of the user’s trousers during walking. The first unit vector, denoted with ζ, is defined by the gravity direction, and is calculated as the mean acceleration within a motion (walking) cycle. The second unit vector, denoted with ξ, is considered to be aligned with the direction of motion and is determined using PCA as the direction with the highest variance once the projection on ζ has been subtracted. The third unit vector, denoted with ψ, is defined as orthogonal to the first two and as such covers lateral motion during the motion cycle.

In our dance analysis context, both the template and subsequence of the best-matching pair represent one full dance move, i.e., motion cycle. Considering this, following the abovementioned, from **A_TM_**, we obtain three orthogonal unit vectors $\zeta_{Τ},\;\xi_{T},\;$ and $\psi_{T}$ while from **A_XM_**, we obtain three orthogonal unit vectors $\zeta_{X},\;\xi_{X},$ and $\psi_{X}.$ Extracting unit vectors ζ, ξ, and ψ from the acceleration 3D projections has already been presented in detail in [20]; we provide details specific to this study in Appendix B.

Combining both triplets of unit vectors, $\zeta_{Τ},\;\xi_{T},\;\psi_{T}$ and $\zeta_{X},\;\xi_{X},\psi_{X},$ we can now define a rotation matrix **R** that aligns the coordinate system of the test sequence with that of the templates as:

(6) $$R=\left[{\begin{array}{ccc} \zeta_{X} & \xi_{X} & \psi \end{array}}_{X}\right]{\left[{\begin{array}{ccc} \zeta_{T} & \xi_{T} & \psi \end{array}}_{T}\right]}^{-1}.$$

Using **R** (6), we can transform the original acceleration and angular velocity 3D projections, comprised in matrices $A_{X}^{}$ and $\Omega_{X}^{},$ respectively, from the test sequence’s device intrinsic to the coordinate system of the template moves. Denoting the resulting matrices with $A_{X}^{\left(R\right)}$ and $\Omega_{X}^{\left(R\right)},$ we write:

(7) $$\begin{array}{l} A_{X}^{\left(R\right)}=A_{X}^{}R\\ \Omega_{X}^{\left(R\right)}=\Omega_{X}^{}R. \end{array}$$

The common *N_X_* × 6 matrix of the rotated acceleration and angular velocity 3D projections of the test sequence, denoted with $X^{\left(R\right)},$ given in the coordinate system of the templates, is then:

(8) $$X^{\left(R\right)}=\left[\begin{array}{cc} A_{X}^{\left(R\right)} & \Omega_{X}^{\left(R\right)} \end{array}\right].$$

#### 2.5.4. Final Template Matching

We can now search the test sequence for template moves, following a similar procedure as presented in Section 2.5.2., only this time considering the acceleration and angular velocity 3D projections instead of their magnitudes. For the template moves and the test sequence, these are comprised in matrices $T_{i}$(2) and $X^{\left(R\right)}$ (6), respectively.

For each template move 1 ≤ *i* ≤ *I* = 15, we first standardise to zero mean and unit standard deviation the columns of $T_{i}$. Vectorising the standardised matrices column-wise, for each template move *i*, we obtain a 6*N*_T_-long vector of acceleration and angular velocity projections **t***_i_*.

We further use a 2D sliding window of size *N*_T_ × 6, and for each 1 ≤ *n* ≤ *N_X_* − *N*_T_ + 1, we extract an *N*_T_ × 6 submatrix of $X^{\left(R\right)},$ i.e., $X^{\left(R\right)}{}_{n:n+N_{T}+1,*}.$ After standardisation and vectorisation, we obtain a 6*N*_T_-long vector of the associated acceleration and angular velocity projections. We then calculate the correlation coefficient between this vector and **t***_i_*.

Sliding the 2D window vertically, we repeated this process for all *n* and obtain *N_X_* − *N*_T_ + 1 correlation coefficients. We store the results for each template move *i* in a vector denoted with $r_{T_{i}X^{\left(R\right)}}.$ This calculation is supported in detail with implementation equations in Appendix A.6.

Indicating high similarity between $T_{i}$ and $X^{\left(R\right)}$, local maximums of $r_{T_{i}X^{\left(R\right)}}$ reveal the possible presence of move *i* in the test sequence. For each dance move *i*, we identify the local maximums of $r_{T_{i}X^{\left(R\right)}}$ that exceed a certain threshold value, *r_min_*. We define the indices of these local maximums as move *i* onsets in the sequence and the associated $r_{T_{i}X^{\left(R\right)}}$ values as the recognition confidence.

After the entire sequence has been searched through, we performed a final correction for false positives detection and elimination: if any two onsets were closer than 0.7 *N*_T_, we discarded the one with the lower recognition confidence.

### 2.6. Recognition Performance Assessment

All performances of dancers for all test sequences were visually inspected by the professional using the accompanying videos. All moves determined to be improperly performed were discarded from analysis. All proper performances were appropriately labelled as one of the 15 moves and considered for assessing the recognition ability of the presented method.

The presented recognition method is non-binary and we assess its recognition ability by considering each move that is correctly recognised as a true positive (TP). Each move that is incorrectly recognised we consider as a false positive (FP). Using cumulative TP and FP rates for all *I* = 15 moves, we calculate the sensitivity and precision scores for each dance tempo considered. We calculate these scores for threshold values *r_min_* ranging from 0.25 to 0.80 with 0.05 increment steps. We finally use the *F1* score, calculated from sensitivity and precision, as the unified measure of recognition ability.

In the specific dance motion recognition context, the sensitivity scores are mainly influenced by the similarity between the analysed moves and the corresponding templates in the database. As such, the sensitivity scores obtained for the professional dancer indicate how consistent her performance is, while those obtained for the recreational dancers show how well they imitate the professional.

Precision scores are additionally influenced by the similarities between different moves, indicated by nondiagonal elements of $r_{\bar{\bar{T_{i}}}\bar{\bar{T_{j}}}}$ for the initial template matching and of $r_{T_{i}T_{j}}$ for the final template matching. Performed on acceleration and angular velocity 3D projections, final template matching is expected to have a higher discriminative potential than the initial template matching, performed using the acceleration and angular velocity magnitudes.

## 3. Results and Discussion

### 3.1. Database of Template Moves

**Figure 2:**
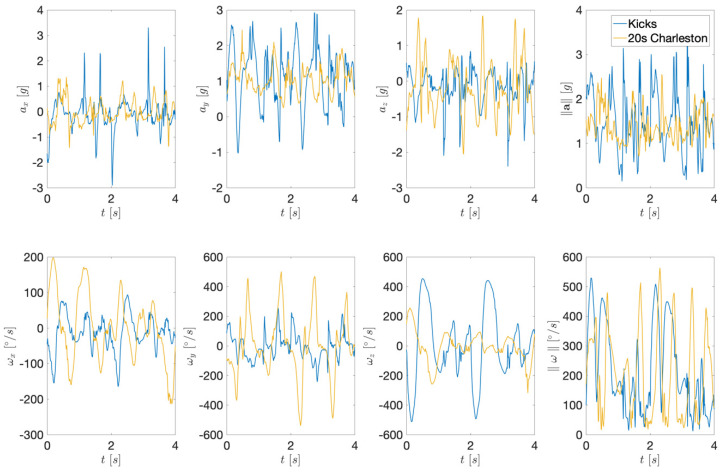
Examples of acceleration (first row) and angular velocity (second row) of right leg motion during two solo jazz moves. The first three columns show the 3D projections and the last column illustrates the magnitudes.

Figure 2 shows the acquired acceleration and angular velocity signals for two illustrative template moves: (3) Kicks and (7) 20s Charleston. The first row depicts the acceleration signals, while the second row shows the angular velocities. Even from pure visual inspection, we can see that both the 3D projections and magnitudes have distinctive signal shapes. It is therefore reasonable to assume that these signal shapes can be used as fingerprints enabling dance move recognition.

The similarities between different template moves $r_{\bar{\bar{T_{i}}}\bar{\bar{T_{j}}}}$, calculated using the magnitudes of acceleration and angular velocity, are presented in Table 1. As expected, some dance moves show greater pairwise similarity while others are more idiosyncratic and show greater distinction from the rest. The highest similarity is $r_{\bar{\bar{T_{7}}}\bar{\bar{T_{8}}}}=0.61,$ obtained for the pair of moves (7) 20s Charleston and (8) Knee slaps, reflecting a leg activation pattern these two moves have in common. Both of these moves are executed mainly with the right leg during the first four music beats and with the left leg during the second four music beats.

The move with the lowest similarity with respect to all other moves, i.e., with the lowest maximum $r_{\bar{\bar{T_{i}}}\bar{\bar{T_{j}}}}$ value, is Apple Jacks (10): $r_{\bar{\bar{T_{10}}}\bar{\bar{T_{j}}}}\leq 0.37$ for 1 ≤ *j* ≤ 15, *j* ≠ 10.

The presented values indicate the discriminative potential of using the acceleration and angular velocity magnitudes for recognising different solo jazz dance moves. In particular, for threshold values *r_min_* > 0.61, high precision scores are expected; however, the higher *r_min_* is, the lower is the expected sensitivity, diminishing the model’s overall recognition ability.

The similarities between different template moves $r_{T_{i}T_{j}},$ calculated using the 3D projections, are presented in Table 2. We can see that these values are generally lower than the corresponding values of $r_{\bar{\bar{T_{i}}}\bar{\bar{T_{j}}}}$, and for all pairs of moves, $r_{T_{i}T_{j}}$ is below 0.5.

The highest $r_{T_{i}T_{j}}$ value is obtained for the pair of moves (4) Half break and (11) Boogie back: $r_{T_{4}T_{11}}=0.43.$ The lowest maximum $r_{T_{i}T_{j}}$ value is 0.24 and is obtained for moves (8) Knee slaps and (14) Cross step. The presented values indicate the expected performance of the final template matching: for threshold values *r_min_* > 0.43, high precision scores are expected.

Lower values of $r_{T_{i}T_{j}}$ obtained with respect to $r_{\bar{\bar{T_{i}}}\bar{\bar{T_{j}}}}$ confirm the natural assumption that comparing dance moves using the 3D projections of signals instead of their magnitudes, considering the direction of motion instead of only the general leg activation pattern, has a higher discriminative potential. Therefore, template matching achieved using the 3D projections instead of magnitudes is expected to provide for higher precision scores, better overall recognition ability, and lower values of *r_min_*.

### 3.2. Dance Move Recognition

For all 36 test sequences, the estimated dance tempo is accurate up to 1 bpm, allowing us to temporally scale all sequences and perform correlation-based template matching, as presented in the previous section.

#### 3.2.1. Validation Using the Professional Dancer’s Test Sequences

**Figure 3:**
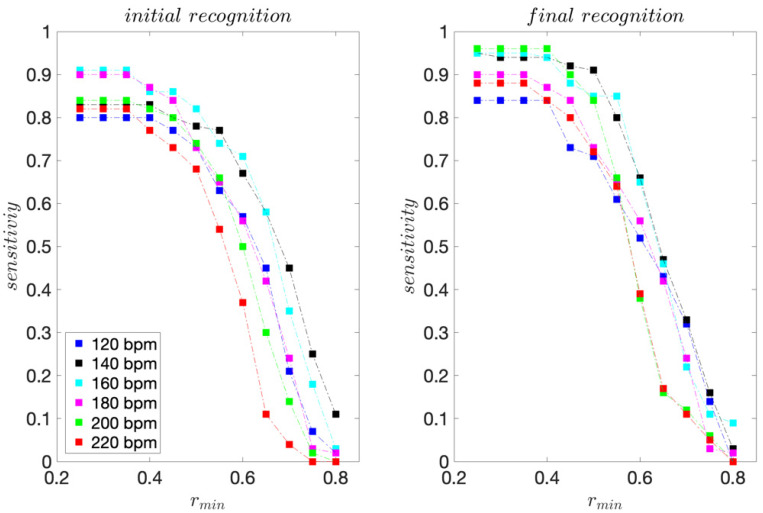
Sensitivity scores obtained for the professional dancer’s test sequences.

**Figure 4:**
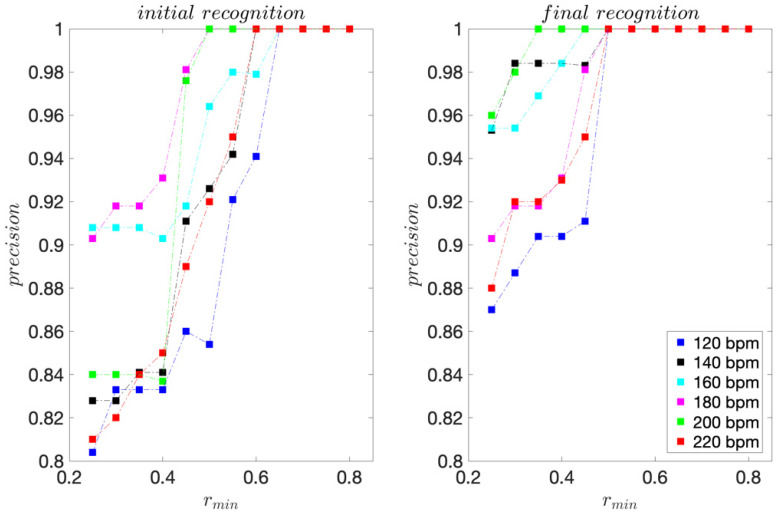
Precision scores obtained for the professional dancer’s test sequences.

For the professional dancer, for each dance tempo 120–200 bpm, 75 moves were acquired. For tempo 220 bpm, 70 moves were acquired. The sensitivity and precision scores are presented in Figure 3 and Figure 4.

For the initial template matching, the highest sensitivity scores range from 0.80 to 0.91 and are obtained for *r_min_* ≤ 0.4. For the final template matching, they range from 0.84 to 0.96 and are also obtained for *r_min_* ≤ 0.4.

For all dance tempos considered, the precision scores are equal to 1 for *r_min_* ≥ 0.65 for the initial template matching and *r_min_* ≥ 0.50 for the final template matching, indicating that for *r_min_* this high, there are no FPs. These results are consistent with the template moves’ pair-wise similarities presented in Table 1 and Table 2. The improvement in precision for the final template matching is a direct consequence of using the 3D projections of the inertial signals instead of their magnitudes for template matching. As expected, considering the direction of motion provides for a better discrimination of moves.

**Figure 5:**
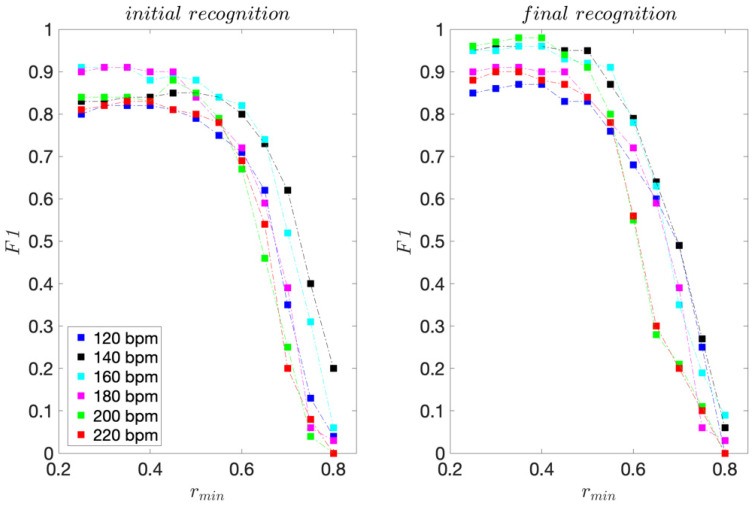
*F1* scores obtained for the professional dancer’s test sequences.

Figure 5 shows the *F1* scores. For each dance tempo considered, the maximum *F1* score indicates the best recognition ability. Lower *F1* scores to the left of the maximum indicate higher FP rates and consequently lower precision. Lower *F1* scores to the right of the maximum indicate lower TP rates and consequently, lower sensitivity.

For the initial template matching, *F1* maximums are obtained for *r_min_* ≤ 0.50. The highest maximum is 0.91, obtained for tempos 160 and 180 bpm for *r_min_* = 0.30 and 0.35. The lowest maximum is 0.82, obtained for the slowest tempo considered, i.e., 120 bpm, for 0.30 ≤ *r_min_* ≤ 0.40. We can also observe that for *r_min_* > 0.60, *F1* scores drop sharply.

Using the criteria of the highest minimum *F1* score at a specific *r_min_*, we can conclude that the overall best recognition performance is achieved for *r_min_* = 0.30 and 0.35, for which the F1 scores range from 0.82 for 120 bpm to 0.91 for 180 bpm dance tempos.

As expected, the *F1* scores for the final template matching exceed those obtained for the initial template matching. The *F1* maximums range from 0.87 to 0.98. The overall best recognition performance is obtained for *r_min_* = 0.35 and 0.40.

These results confirm the potential of using the presented method to recognise repeated dance moves and to assess dancers’ consistency in performance.

#### 3.2.2. Validation Using the Recreational Dancer’s Test Sequences

**Figure 6:**
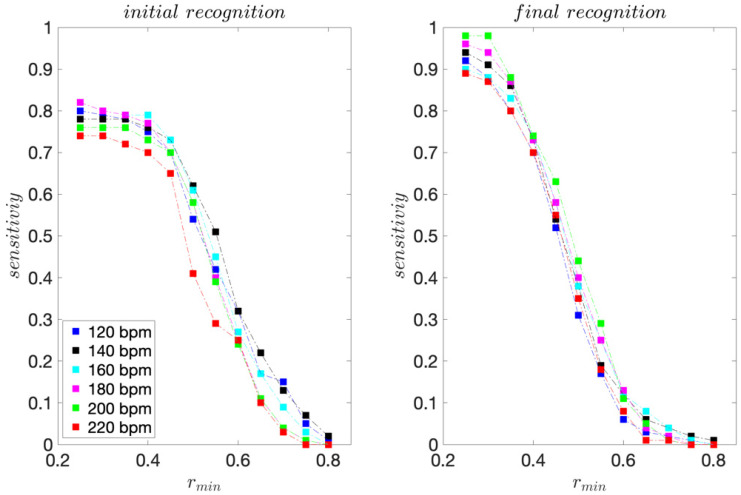
Sensitivity scores obtained for the recreational dancers’ test sequences.

**Figure 7:**
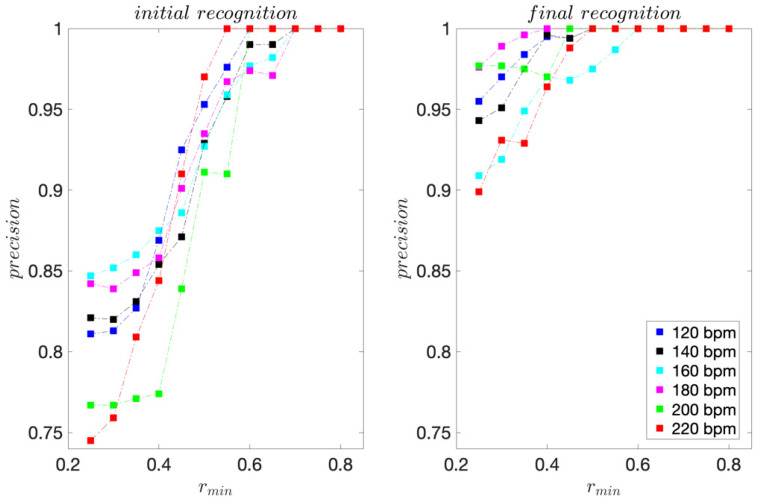
Precision scores obtained for the recreational dancers’ test sequences.

**Figure 8:**
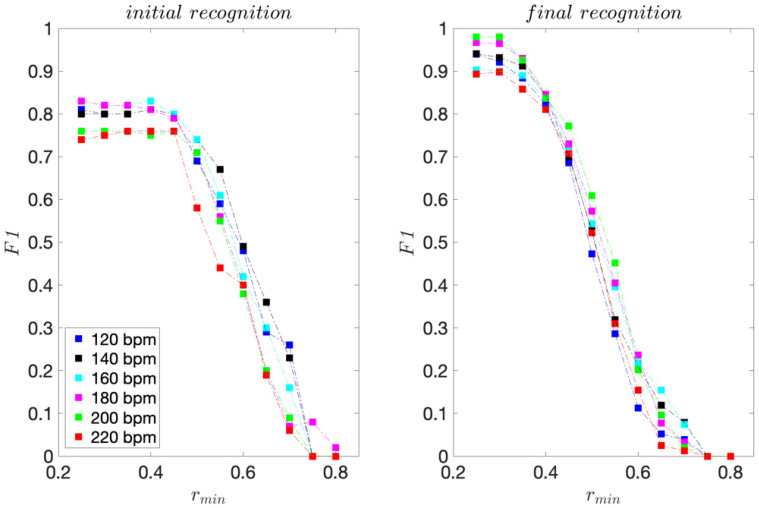
*F1* scores obtained for the recreational dancers’ test sequences.

For the recreational dancers, 293–311 dance moves were collected for each dance tempo considered. The sensitivity, precision, and *F1* scores are presented in Figure 6, Figure 7 and Figure 8, respectively.

For the initial template matching, the sensitivity maximums range from 0.74 to 0.82 and are obtained for *r_min_* ≤ 0.40. For the final template matching, they range from 0.89 to 0.98 and are obtained for *r_min_* ≤ 0.30. For all dance tempos considered, the precision scores are equal to 1 for *r_min_* ≥ 0.70 for the initial template matching and *r_min_* ≥ 0.60 for the final template matching.

For the initial template matching, the *F1* maximums range from 0.76 to 0.84 and are obtained for *r_min_* ≤ 0.45. For the final template matching, the *F1* maximums range from 0.89 to 0.98 and are obtained *r_min_* ≤ 0.30.

The main difference when compared to the professional dancer’s results is that the maximum *F1* scores are obtained for lower values of *r_min_*. In addition, after rising to their maximum value, *F1* scores fall more sharply. These observations are an expected consequence of individual style and subtle differences in move performances of different dancers.

The results confirm the potential of using the presented method to recognise imitated dance moves. Similar recognition ability can be achieved for the template professional dancer, but at lower values of *r_min_*.

Finally, the recognition ability of the final template matching proves that extracting three device-independent coordinate system axes from the acquired dance motion acceleration signals as presented in the previous section is valid and enables reliable transformation of the inertial signals from the device-intrinsic to the coordinate system of the templates.

## 4. Conclusions

We have shown that a single wearable device, capturing 3D acceleration and angular velocity of a dancer’s right leg motion, provides for recognising 15 solo jazz dance moves. We have demonstrated that the analysed sequences can be reliably temporally scaled and that dance recognition can be achieved using the same templates, independent of the tempo of dancing. Considering this makes the creation of the template database easier.

Residing on a single device makes the solution highly practical. The practical aspect is further enhanced with the recognition’s independency of the device orientation. The results confirm that even for such a dynamic motion as solo jazz dancing, the acquired acceleration and angular velocity 3D projections can be reliably transformed to a device orientation-invariant system. Exploiting the 3D projections of the signals has a higher discriminative potential and provides for better recognition ability than does considering only the signals’ magnitudes.

The presented solution fills the gap of the existing state of the art: since the presented solution avoids using video cameras or IR imaging sensors, it is not computationally expensive or limited to specific locations; residing on a single wearable device and not being dependent on the orientation of the wearer makes the methodology suitable for unsupervised everyday use and applicable for a variety of dancing situations—whether the dancer is dancing alone, in the crowd, or in front of an audience.

Activity recognition methods relying on wearable devices, developed for ubiquitous measurement scenarios, usually try to balance the opposing requirements of device life autonomy and recognition performance. By relying on a limited set of features extracted from the inertial signals, these methods reduce the computational complexity and prolong the device life autonomy. Since the methodology presented in this article is primarily intended to be used for relatively short dancing sequences, e.g., per song basis, as opposed to throughout-the-day use, more computational power, needed for performing the correlation-based template matching, can be invested in the analysis.

The advantages for the dancer and/or dancing instructor are straightforward: for each performed dance sequence, dance moves can be extracted together with recognition confidence levels, indicating how well the execution matches the reference template. This way, the methodology supports progress monitoring. In addition, the recognised moves can be further analysed and compared to the reference moves in the database to evaluate the overall performance. Finally, while tested on solo jazz dance moves, the methodology presented in this article can be extended to other dance styles, as well.

The presented methodology demonstrates a high recognition ability for the preliminary set of measurements obtained for six dancers. However, a standalone study is needed to further explore in-depth the variability between dancers. Further analysis is also necessary to assess the methodology recognition performance in real dancing scenarios, when the dancer is dancing freely, combining various predefined and improvised moves in sequences. Further studies can also capitalise on this study and assess the dancer’s creativity and response to music, investigating the crucial connection between dancing and music. The benefits of using additional devices, positioned on other body segments, can be investigated. Finally, the presented implementation can be explored further with the aim of optimisation for real-time execution.

## Figures and Tables

**Table 1:** Solo jazz template moves’ pairwise similarities calculated using the magnitudes of acceleration and angular velocity. Depicted in red are the highest similarities obtained for each move, excluding self-similarity.

Move	(1)	(2)	(3)	(4)	(5)	(6)	(7)	(8)	(9)	(10)	(11)	(12)	(13)	(14)	(15)
(1)	1.00	0.22	0.45	0.29	0.39	0.29	0.37	0.27	0.35	0.35	0.32	0.46	0.27	0.31	0.31
(2)		1.00	0.19	0.21	0.35	0.47	0.29	0.44	0.34	0.28	0.33	0.33	0.27	0.30	0.37
(3)			1.00	0.42	0.39	0.28	0.30	0.33	0.35	0.26	0.36	0.55	0.33	0.43	0.30
(4)				1.00	0.37	0.29	0.31	0.36	0.39	0.24	0.43	0.55	0.32	0.27	0.43
(5)					1.00	0.33	0.37	0.37	0.29	0.26	0.29	0.48	0.23	0.48	0.38
(6)						1.00	0.38	0.42	0.33	0.32	0.44	0.38	0.24	0.36	0.44
(7)							1.00	0.61	0.20	0.37	0.31	0.32	0.25	0.33	0.28
(8)								1.00	0.27	0.34	0.47	0.39	0.34	0.30	0.42
(9)									1.00	0.22	0.29	0.24	0.30	0.43	0.31
(10)										1.00	0.29	0.35	0.31	0.27	0.30
(11)											1.00	0.47	0.54	0.26	0.35
(12)												1.00	0.39	0.31	0.43
(13)													1.00	0.19	0.32
(14)														1.00	0.43
(15)															1.00

**Table 2:** Solo jazz template moves’ pairwise similarities calculated using the 3D projections of the acceleration and angular velocity. Depicted in red are the highest similarities obtained for each move, excluding self-similarity.

Move	(1)	(2)	(3)	(4)	(5)	(6)	(7)	(8)	(9)	(10)	(11)	(12)	(13)	(14)	(15)
(1)	1.00	0.18	0.28	0.21	0.23	0.15	0.23	0.15	0.26	0.32	0.25	0.19	0.10	0.23	0.25
(2)		1.00	0.22	0.10	0.22	0.23	0.20	0.23	0.12	0.21	0.15	0.22	0.14	0.29	0.19
(3)			1.00	0.21	0.40	0.18	0.25	0.10	0.19	0.186	0.18	0.32	0.13	0.38	0.18
(4)				1.00	0.26	0.19	0.13	0.12	0.26	0.17	0.43	0.34	0.11	0.16	0.40
(5)					1.00	0.23	0.35	0.14	0.17	0.20	0.26	0.33	0.10	0.34	0.25
(6)						1.00	0.33	0.22	0.17	0.18	0.18	0.16	0.13	0.23	0.20
(7)							1.00	0.21	0.11	0.23	0.25	0.15	0.12	0.38	0.26
(8)								1.00	0.23	0.15	0.11	0.17	0.09	0.24	0.10
(9)									1.00	0.19	0.19	0.30	0.20	0.15	0.26
(10)										1.00	0.18	0.21	0.29	0.17	0.22
(11)											1.00	0.28	0.23	0.14	0.39
(12)												1.00	0.18	0.27	0.29
(13)													1.00	0.12	0.18
(14)														1.00	0.19
(15)															1.00

## Data Availability

The data presented in this study are available on request from the corresponding author.

## Appendix A

### Appendix A.1. Signal Matrices Similarity

Let **M** be an arbitrary *N* × *K* matrix. Let $\overset{⌢}{M}$ further be a matrix with columns equal to the columns of matrix **M**, standardised to zero mean and unit standard deviation:

(A1) $${\overset{⌢}{M}}_{*,k}=\frac{M_{*,k}-\mu_{M_{*,k}}}{\sigma_{M_{*,k}}},\;1\leq k\leq K,$$

where μ and σ denote the mean and standard deviation, respectively, while * denotes all rows.

Let now **m** be an *LK*-long vector obtained by vectorising matrix $\overset{⌢}{M}$ column-wise:

(A2) $$m={\left[\begin{array}{cccc} {\overset{⌢}{M}}_{*,1}{}^{T} & {\overset{⌢}{M}}_{*,2}{}^{T} & \ldots & {\overset{⌢}{M}}_{*,K}{}^{T} \end{array}\right]}^{T}.$$

In (A2),${}^{T}$ denotes the transpose operator.

Following (A1) and (A2), for any two matrices **M** and **N** of equal size *N* × *K*, we can now define a similarity measure $r_{MN},$ calculated as the correlation coefficient across the matrices’ *K* dimension as:

(A3) $$r_{MN}=m^{T}n/(NK).$$

In particular, instead of **M** and **N**, we use signal matrices with columns representing 1 ≤ *k* ≤ *K* signals and rows representing 1 ≤ *n* ≤ *N* time samples. The calculated similarity measure (A3) represents the correlation coefficient between two sets of *K* signals.

### Appendix A.2. Dance Move Repetition Extraction

Let **S** be an *N**_s_* × 6 signal matrix, with columns representing the 6 inertial signals and rows representing the signals’ *N_s_* time samples. For a defined starting row *n_0_* and an initial length *N_n_*, let us extract two adjacent *N_n_* × 6 submatrices of **S**. By standardising these two submatrices according to (A1) and applying (A2), we obtain their respective vector forms. Further applying (A3), we obtain the correlation coefficient, indicating the submatrices’ similarity.

Repeating the aforementioned process for different lengths of the submatrices, *N_n_* = *N_0_* + *n*, where, in particular for the problem at hand, *N_0_* = 392 and 0 ≤ *n* ≤ 16, we obtain a vector of correlation coefficients **r_S_**. Finally, by identifying the index of the **r_S_** maximum, denoted with *n_max_*, we obtain the number of rows *N_nmax_*:

(A4) $$N_{nmax}=N_{0}+n_{max}$$

for which the adjacent submatrices match the most. These two submatrices, i.e., $S_{n_{0}:}{{}_{n_{0}+N_{nmax}-1}}_{,*}$ and $S_{n_{0}+N_{nmax}:n_{0}+2N_{nmax}-1}{}_{,*},$ are used as signal matrices of two consecutive repetitions of a dance move.

### Appendix A.3. Magnitudes Similarity

Let $\bar{\bar{T_{i}}}\;\mathrm{and}\;\bar{\bar{T_{j}}}\;$ denote *N*_T_ × 2 matrices of acceleration and angular velocity magnitudes for template moves *i* and *j*. By inserting $\bar{\bar{T_{i}}}$ instead of **M** in (A1)–(A2), we perform matrix normalisation and vectorisation. We denote the resulting 2*N*_T_-long vector with $\bar{\bar{t_{i}}}.\;$

For each 0 ≤ *τ* < *N*_T_, we cyclically shift matrix $\bar{\bar{T_{j}}}\;$ rows:

(A5) $${\bar{\bar{T_{j}}}}_{\tau }=\left[\begin{array}{c} \bar{\bar{T_{j}}}{\;}_{\tau +1:N_{T},*}\\ {\bar{\bar{T_{j}}}}_{1:\tau ,*} \end{array}\right].$$

By inserting ${\bar{\bar{T_{j}}}}_{\tau }\;$ instead of **M** in (A1)–(A2), we perform normalisation and vectorisation. For each *τ*, we denote the resulting 2*N*_T_-long vector with ${\bar{\bar{t_{j}}}}_{\tau }.$ By further inserting $\bar{\bar{t_{i}}}$ and ${\bar{\bar{t_{j}}}}_{\tau }$ instead of **m** and **n** in (A3) for each *τ*, we calculate a component of the similarity vector, denoted with $r_{\bar{\bar{T_{i}}}\bar{\bar{T_{j}}}}.$ We use the maximum value of this vector, denoted with $r_{\bar{\bar{T_{i}}}\bar{\bar{T_{j}}}}$, as the two moves’ magnitude similarity measure.

### Appendix A.4. Projections Similarity

Let **T***_i_* and **T***_j_* denote two *N*_T_ × 6 matrices of 3D acceleration and angular velocity projections for template moves *i* and *j*. By inserting **T***_i_* instead of **M** in (A1)–(A2), we perform normalisation and vectorisation. We denote the resulting 6*N*_T_-long vector with $t_{i}.$

For each 0 ≤ *τ* < *N**_T_*, we cyclically shift matrix **T***_j_* rows:

(A6) $$T_{j}{}_{\tau }=\left[\begin{array}{c} T_{j}{\;}_{\tau +1:N_{T},*}\\ T_{j\;}{}_{1:\tau ,*} \end{array}\right].$$

By inserting $T_{j}{}_{\tau }$ instead of **M** in (A1)–(A2), we perform normalisation and vectorisation. For each *τ*, we denote the resulting 6*N*_T_-long vector with $t_{j\;\tau }.\;$ By further inserting $t_{i}$ and $t_{j\;\tau }$ instead of **M** and **N** in (A3) for each *τ*, we calculate a component of the similarity vector, denoted with $r_{T_{i}T_{j}}$. We use the maximum value of this vector, denoted with $r_{T_{i}T_{j}}$, as the two moves’ projection similarity measure.

### Appendix A.5. Initial Template Matching

Let $\bar{\bar{X}}$ denote an *N**_T_* × 2 matrix of the test sequence 3D acceleration and angular velocity magnitudes. For each 1 ≤ *n* ≤ *N_X_* – *N*_T_ + 1, we extract a submatrix of $\bar{\bar{X}},$ i.e., ${\bar{\bar{X}}}_{\;n:n+N_{T}-1,*}.$ By inserting this submatrix instead of **M** in (A1)–(A2), we perform column-wise standardisation and matrix vectorisation, obtaining a 2*N*_T_-long vector denoted with ${\bar{\bar{x}}}_{n}$ for each *n*.

With the signals prepared, we can now calculate the vector of correlation coefficients between the magnitude signals of the sequence and the templates. For each template move *i*, we denote this vector with $r_{\bar{\bar{T_{i}}}\bar{\bar{X}}}.$ For each time lag *n*, we obtain an element of vector $r_{\bar{\bar{T_{i}}}\bar{\bar{X}}}$ by inserting $\bar{\bar{t_{i}}}$ and $\bar{\bar{x_{n}}}$ instead of **M** and **N** in (A3):

(A7) $$r_{\bar{\bar{X}}\bar{\bar{T_{i}}}}(n)={\bar{\bar{t_{i}}}}^{T}\bar{\bar{x_{n}}}/(2N_{T}).$$

Indicating high similarity between $\bar{\bar{T_{i}}}\;\mathrm{and}\;\bar{\bar{X}}$, local maximums of $r_{\bar{\bar{T_{i}}}\bar{\bar{X}}}$ reveal the possible onsets of template move *i* executions in the test sequence.

### Appendix A.6. Final Template Matching

Let $X^{\left(R\right)}$ denote an *N*_T_ × 6 matrix of the test sequence 3D accelerations and angular velocities projections on the templates’ coordinate system axes. For each time lag 1 ≤ *n* ≤ *N_X_* − *N*_T_ + 1, we extract a submatrix of $X^{\left(R\right)},$ i.e., $X^{\left(R\right)}{}_{\;n:n+N_{T}-1,*}.$ By inserting this submatrix instead of **M** in (A1)–(A2), we perform column-wise standardisation and matrix vectorisation, obtaining a 6*N*_T_-long vector, denoted with $x_{n}^{\left(R\right)}$ for each *n*.

We can now calculate the vector of the correlation coefficients between the projection signals of the sequence and the templates. For each template move *i*, we denote this vector with $r_{T_{i}X^{\left(R\right)}}.$ For each time lag *n*, we obtain an element of $r_{T_{i}X^{\left(R\right)}}$ by inserting $t_{i}$ and $x_{n}^{\left(R\right)}$ instead of **M** and **N** in (A3):

(A8) $$r_{T_{i}X^{\left(R\right)}}(n)=t_{i}{}^{T}x_{n}^{\left(R\right)}/(6N_{T}).$$

Indicating high similarity between $T_{i}$ and $X^{\left(R\right)},$ local maximums of $r_{T_{i}X^{\left(R\right)}}$ reveal the possible presence of move *i* execution in the test sequence.

## Appendix B

### Orientation Independent Transformation

Let **A** be an *N*_T_ × 3 matrix of 3D acceleration signals of a single dance move. The columns of **A** represent the acceleration projections on the device-intrinsic coordinate system axes and rows represent the time dimension. Following the procedure presented in [20], we extract three orthogonal 3 × 1 unit vectors from **A** as follows.

The orientation of the first unit vector, denoted with ζ, is defined as the gravity direction, being parallel to the user’s torso, and is calculated as the mean acceleration direction within the motion cycle, i.e., dance move. Denoting a as the mean direction of acceleration during one dance move, we can write:

(A9) $$\begin{array}{c} a={\left[\begin{array}{ccc} \mu_{A_{*,1}} & \mu_{A_{*,2}} & \mu_{A_{*,3}} \end{array}\right]}^{T}\\ \zeta =a/\left\|a\right\|. \end{array}$$

In (A9), μ and ‖‖ denote the vector mean and magnitude calculation, respectively, while ^T^ denotes the transpose operator. Acceleration projection on axis ζ is then calculated:

(A10) $$A_{\zeta }=A\zeta$$

and subtracted from the original 3D acceleration signals, giving acceleration in the plane orthogonal to the gravity direction:

(A11) $$A_{\zeta }^{f}=A-\zeta A_{\zeta }{}^{T}.$$

The orientation of the second unit vector ξ is defined as the direction covering most of $A_{\zeta }^{f}$ variance. Normalised eigenvectors of $A_{\zeta }^{f}$ are determined using Principal Component Analysis (PCA). The one associated with the maximum eigenvalue is set as ξ. Finally, the remaining unit vector is defined as being orthogonal to the first two:

(A12) ψ=ζ×ξ.

In (A12), x denotes the cross product.

We apply this method to our dance signals. By inserting the 3D acceleration signal matrices of the template and subsequence of the best matching pair, **A_TM_** and **A_XM_**, respectively, instead of **A** in (A9)–(A12), we obtain two triplets of orthogonal unit vectors. From **A_TM_**, we obtain unit vectors $\zeta_{T},\;\xi_{T},\;\mathrm{and}\;\psi_{T}$, while from **A_XM_**, we obtain three orthogonal unit vectors $\zeta_{X},\;\xi_{X},\;\mathrm{and}\;\psi_{X}.$
